# Prediction of effective Lens position using anterior segment optical coherence tomography in Chinese subjects with angle closure

**DOI:** 10.1186/s12886-021-02213-w

**Published:** 2021-12-27

**Authors:** Yuzhou Wu, Shunhua Zhang, Yong Zhong, Ailing Bian, Yang Zhang, Zaowen Wang

**Affiliations:** 1 Department of Ophthalmology, Peking Union Medical College Hospital, Chinese Academy of Medical Sciences, No.1, Shuai Fu Yuan street, Dong Cheng district, Beijing, 100730 China; 2 Key Laboratory of Ocular Fundus Diseases, Chinese Academy of Medical Sciences & Peking Union Medical College, Beijing, China

**Keywords:** Primary angle closure Glaucoma, Anterior segment optical coherence tomography, Formula, Effective Lens position

## Abstract

**Purpose:**

To assess the accuracy of biometric parameters measured by anterior segment optical coherence tomography (AS-OCT) and partial coherence interferometry (PCI) in prediction of effective lens position (ELP) compared with previous formulas in PACG patients.

**Methods:**

121 PACG eyes were randomly divided into training set (85 eyes) and validation set (36 eyes) with same procedure including AS-OCT, PCI, phacoemulsification and IOL implantation surgery. Preoperative anterior chamber depth (pre-ACD), scleral spur depth (SSD), scleral spur width (SSW), lens vault (LV) and cornea thickness (CT) were measured from AS-OCT image. Axial length (AL) and corneal power (K) were measured by PCI. All the 7 parameters were analyzed by multiple linear regression in training set and a statistic regression formula was developed. In validation set, one-way ANOVA was applied to compare the new regression formula with Sanders-Retzlaff-Kraff theoretic (SRK/T), Holladay 1, Haigis, and a regression formula developed in previous study.

**Results:**

The coefficient of determination (R^2^) of different parameter combinations are 0.19 (pre-ACD, AL), 0.25 (AL, K) and 0.49 (SSD, AL, SSW) in training set. In validation set, the correlation between predicted and measured ELP are: new formula (R^2^ = 0.50, *P* = 0.9947) Holladay 1 (R^2^ = 0.12, *P* < 0.0001), SRK/T (R^2^ = 0.11, *P* < 0.0001) and Haigis (R^2^ = 0.06, *P* < 0.0001).

**Conclusion:**

Among 7 tested parameters, pre-ACD contribute little in ELP prediction. Formula consist of SSD, AL and SSW showed better accuracy than other formulas tested.

## Introduction

Primary Angle Closure Glaucoma (PACG) is an disastrous eye disease, leading to permanent optic neuropathy, visual field impairment and irreversible blindness in millions of people worldwide [1, 2]. PACG is the major form of glaucoma and the most important reason of bilateral blindness in Asia. Early diagnosis and early treatment are essential for PACG patients.

In recent years, lens extraction has been widely accepted as the preferred surgical treatment of PACG [3, 4]. With deeper postoperative anterior chamber and lower postoperative intraocular pressure (IOP), PACG patients could benefit from phacoemulsification and intraocular lens (IOL) implantation [3, 5]. Moreover, clear-lens extraction has been proved effective to reduce the risk of acute angle closure [4]. With the development of surgical technique and IOL implantation, it is possible to introduce multifocal IOL (MIOL) into the treatment for early-stage PACG. Lens extraction with MIOL implantation could not only eliminate the risk of acute angle closure, but also correct hypermetropia and presbyopia in Primary Angle Closure Suspect (PACS) and Primary Angle Closure (PAC) patients without manifest damage [4]. This treatment improves quality of life in early-stage PACG patients but require high accuracy of IOL calculation.

However, PACS, PAC and PACG patients have various anatomical crowding including short axial length (AL), disproportionally short anterior chamber, thick lens, small white-to-white distance, etc. [6]. As a result, using available IOL calculation formulas to predict postoperative refractive error in PACG patients is inaccurate, even with the latest generation [6–10]. It is worth improving the accuracy of IOL power calculation in PACG patients.

The major factor which cause refractive prediction error in PACG is inaccurate prediction of effective lens position (ELP). ELP is an objective parameter directly relate to IOL calculation, despite IOL type, power and formula. Consequently, ELP is more reliable than postoperative refractory error to be predicted. The 3rd generation formulas use AL and corneal power (K) to increase the accuracy of ELP prediction [11–13]; while 4th generation formulas such as Haigis and Barrett Universal II use preoperative anterior chamber depth (ACD) and AL for ELP prediction [14]. Preoperative ACD significantly increase the accuracy of ELP prediction in patients who have spacious anterior chamber. However, anterior segment varies in anatomy due to individual differences among PACG patients, leaving postoperative ACD unpredictable [3]. Consequently, preoperative ACD may have less significance or even may be the source of biases deviation in PACGs.

Anterior Segment-Optical Coherence Tomography (AS-OCT) is an useful device to assess anterior segment. It could not only show the crowded condition of anterior chamber but also provide biometric parameter measurement. Previous studies have reported that lens vault showed great correlation to postoperative refractory error in PAC and PACG patients [15]. Also, new formulas which combined partial coherence interferometry (PCI) with AS-OCT have reduced the prediction error of ELP in healthy eyes [16–19]. However, studies on PACG are few. It is promising to find a method to predict ELP more accurately with AS-OCT in PACG patients.

This study is aimed to develop a new formula for PACG patients; minimalize prediction error of ELP and therefore enable PACS and early-stage PAC patients to be implanted with multifocal IOL.

## Methods

### Patients

This is a retrospective case series research. Consecutive patients underwent phacoemulsification lens extraction and IOL implantation with or without goniosynechialysis in ophthalmology department of Peking Union Medical College Hospital were enrolled from December 2013 to August 2019. All surgery were finished by an experienced surgeon (S.Zhang). The inclusion criteria were: (1) Patient had history of unilateral or bilateral, PAC or PACG, with ever elevated IOP (> 30 mmHg); (2) Gonioscope examination confirmed angle closure. Primary angle closure was defined as iridotrabecular contact, either appositional or synechial, of at least 180°on gonioscopy; (3) Patient’s IOP was controlled with or without medication (under 21 mmHg) when captured AS-OCT. The exclusion criteria were: (1) Secondary angle closure, such as glaucoma associated with lens dislocation; (2) Any kind of eye diseases which could affect the assessment of anterior segment and axial length; (3) IOL was dislocated or not implanted in capsular bag; (4) Any postoperative complication such as fluid misdirection syndrome and uveal effusion syndrome; (5) The patient’s preoperative and postoperative data could not be completely retrieved.

This study was approved and supervised by the institutional review board of Peking Union Medical College Hospital in agreement on the declaration of Helsinki. All patients signed informed consent about medical analysis of image data.

### Procedures

According to our workflow of PACG, patient once diagnosed with PAC and PACG subsequently underwent preoperative examinations, phacoemulsification and IOL implantation. Phacoemulsification was performed with a 2.2-mm corneal incision and a three-part IOL (AF-1 YA-60BB, Hoya Corp, Tokyo, Japan) was implanted in the bag under topical anesthesia. Goniosynechialysis was operated on patient who had peripheral anterior synechiae.

### Examinations

Patients underwent slit lamp examination, gonioscopy, fundoscopy and IOL calculation at 1 week before surgery. IOL power was calculated by PCI (IOLMaster 500, version 4.08, Carl Zeiss Meditec, Dublin, California, USA). Patients underwent examination at one room by one experienced technician (Y.Wu). AL and K were read from the examination result. AL reading was average of 10 measures with good consistency.

### AS-OCT data acquisition and processing

Patients underwent anterior segment assessment with AS-OCT (Visante, version 3.0.1.8, Carl Zeiss Meditec, Dublin, California, USA) at 1 week before the surgery and 1 month after surgery. The examination was performed in a dark room (0 lx) by an experienced operator, who was masked to all clinical data. AS-OCT was captured by horizontal scan displaying nasal and temporal quadrants of angle with superluminescent diode 1310-nm light. Each scan was taken 3 times to take an average.

Images of these eyes were extracted for quantitative analysis. Preoperative ACD (pre-ACD), scleral spur distance (SSD), scleral spur width (SSW), lens vault (LV) and corneal thickness (CT) were read from preoperative AS-OCT image in every patient; IOL thickness, postoperative ACD (post-ACD) and ELP were read from postoperative AS-OCT image by one doctor (Y.Wu).

**Fig. 1:**
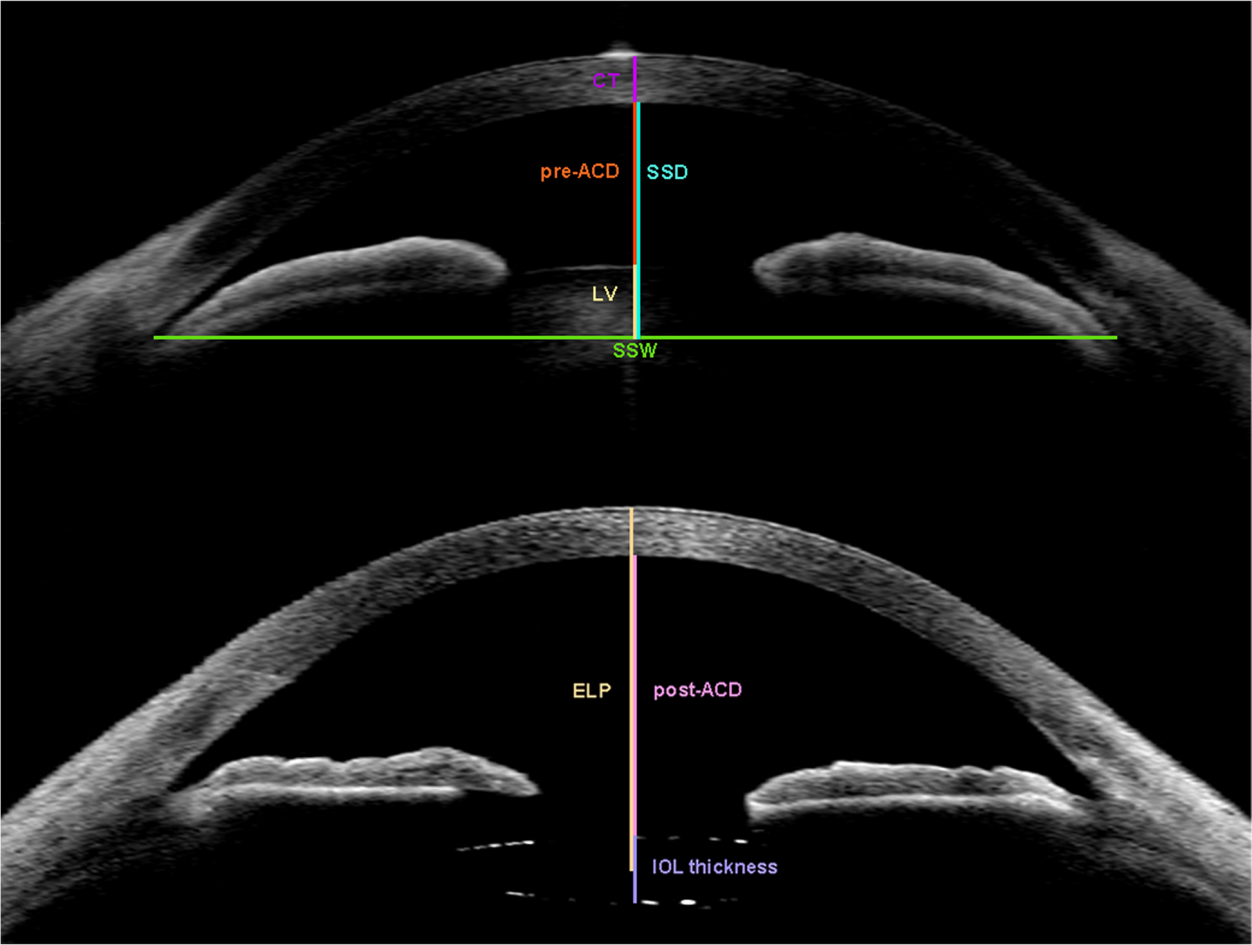
Schematic diagram of the parameters used in the new formula pictured by AS-OCT, CT = corneal thickness; ELP = effective lens position; IOL = intraocular lens; LV = lens vault; post-ACD = postoperative anterior chamber depth; pre-ACD = preoperative anterior chamber depth; SSD = scleral spur distance; SSW = scleral spur width

AS-OCT parameters are defined as follows (Fig. 1): Pre-ACD is defined as the distance between posterior cornea surface and anterior lens surface in preoperative AS-OCT image; and post-ACD is defined as the same distance in postoperative AS-OCT image. SSW is defined as the horizontal scleral spur-to-spur distance; and SSD is defined as the perpendicular distance between the posterior corneal surface and a line drawn between the scleral spur on nasal and temporal sides of the horizontal AS-OCT scans. CT is defined as cornea thickness measured at cornea vertex. LV is defined as the perpendicular distance from anterior lens surface to SSW. ELP is defined as the distance from cornea vertex to IOL center. IOL Thickness is defined as the perpendicular distance from anterior IOL surface to posterior IOL surface. Angle Opening Distance (AOD) is defined as the distance between the point on internal cornea surface a certain length from scleral spur and the opposite point on iris. Scleral Spur Angle (SSA) is defined as the angle formed by trabecular meshwork and the line through scleral spur and the opposite point on iris. Trabecular Iris Space Area (TISA) is defined as the area surrounded by a line drawn from scleral spur to opposing iris, iris surface, AOD and inner corneoscleral wall.

### Statistical analysis

**Fig. 2:**
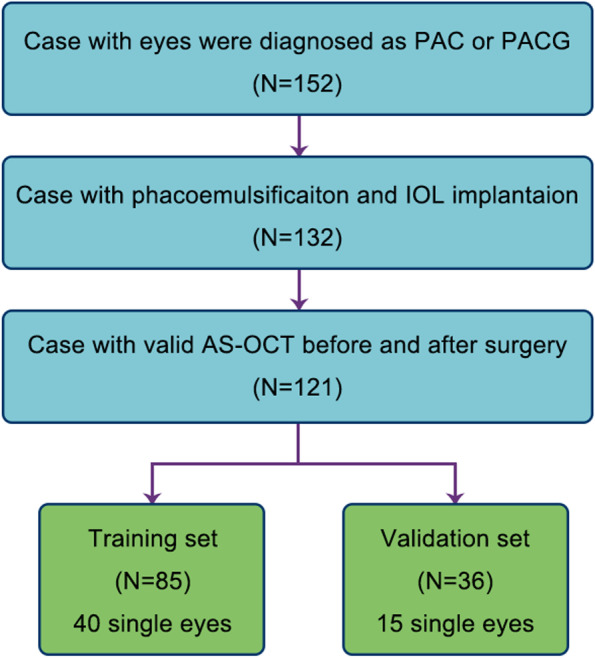
Selection criteria used in this study

All the eyes enrolled in this research were randomly divided into training set and validation set by a ratio of 7:3 (Fig. 2). According to mathematical proof, a 7:3 split of training set and validation set is better than equal division in small sample set, giving consideration to both accuracy and confidence. Demographic data was analyzed through student *t* test and Fisher exact test to testify the random of group division. Seven parameters including pre-ACD, AL, CT, LV, K, SSD and SSW were analyzed to test the correlation with ELP.

In training set, 7 parameters (pre-ACD, AL, CT, LV, K, SSD, SSW) were evaluated by multiple linear regression, among which pre-ACD, CT, LV, SSD and SSW were measured by AS-OCT while AL and K were measured by PCI. Preoperative and postoperative data were analyzed separately in training and validation set by paired *t* test. A new IOL power calculation formula was developed from training set by multiple linear regression (stepwise).

The accuracy of ELP predicted by new formula was compared with 3rd generation formulas (Sanders-Retzlaff-Kraff theoretic [SRK/T], Holladay 1), 4th generation formula (Haigis) and a statistical regression formula using AS-OCT and PCI parameters (marked as 2016 formula) [16] . The predicted ELP was calculated respectively in Holladay 1, SRK/T and Haigis formula, using the following constants: Holladay 1 Surgeon factor = 1.75; SRK/T A-constant = 118.9; Haigis a_0_ = 1.300, a_1_ = 0.400, a_2_ = 0.100. One-way ANOVA and Pearson correlation were used to analyze the difference of ELP prediction between different formulas.

All the statistical data were processed by IBM SPSS Statistics 25 (IBM Corp, Armonk, NY, USA). Figures were drawn by Prism 8 (GraphPad Software Inc., San Diego, California, USA).

## Results

### Baseline clinical characteristics

From December 2013 to August 2019, 152 eyes have been diagnosed as PAC or PACG at Peking Union Medical College Hospital. Phacoemulsification and IOL implantation were done by one surgeon (S.Zhang). No intra-operation complication happened. Eight eyes were excluded because of fluid misdirection syndrome after surgery. Twenty-three eyes were excluded because of incomplete data. One hundred twenty-one eyes with complete data and clearly recognized AS-OCT image could be retrieved, including 55 patients with unilateral eye and 33 patients with bilateral eyes (Fig. 2). All the 121 eyes were randomly divided into training set (85 eyes, among which 40 single eyes) and validation set (36 eyes, among which 15 single eyes). All patients enrolled are Chinese.

### Biometric parameters in training set and validation set

Biometric parameters are measured and analyzed in training set and validation set separately. Demographic data and main measurement outcome in two sets are analyzed (Table 1). All parameters showed no significant difference between training set and validation set.

**Table 1:** Preoperative and Postoperative Measurements in Training and Validation Sets

	Training Set (*N* = 85) Mean (SD)	Validation Set (*N* = 36) Mean (SD)	*P* value
Measures			
Gender (Female: Male)	71:14	27:9	NA
Age (year)	69.38 (9.57)	69.47 (9.35)	0.96
AOD 500 (mm)	0.07 (0.05)	0.08 (0.07)	0.56
AOD 750 (mm)	0.12 (0.07)	0.13 (0.10)	0.76
TISA 500 (mm)	0.03 (0.02)	0.03 (0.02)	0.55
TISA 750 (mm)	0.05 (0.04)	0.06 (0.04)	0.57
SSA (degree)	7.78 (5.58)	8.43 (7.07)	0.60
Pre-ACD (mm)	1.79 (0.30)	1.75 (0.31)	0.49
Post-ACD (mm)	3.49 (0.27)	3.45 (0.27)	0.44
Preoperative SSD (mm)	2.87 (0.22)	2.82 (0.22)	0.20
Postoperative SSD (mm)	2.94 (0.30)	2.88 (0.22)	0.23
Preoperative SSW (mm)	11.19 (0.39)	11.14 (0.37)	0.49
Postoperative SSW (mm)	11.19 (0.39)	11.13 (0.44)	0.44
Preoperative CT (mm)	0.54 (0.05)	0.54 (0.05)	0.36
Postoperative CT (mm)	0.55 (0.06)	0.55 (0.05)	0.44
LV (mm)	1.09 (0.30)	1.05 (0.36)	0.54
AL (mm)	22.31 (0.85)	22.21 (0.94)	0.52
K (diopters)	44.64 (1.60)	44.62 (1.76)	0.94
IOL Thickness (mm)	0.90 (0.14)	0.89 (0.11)	0.63
Measured ELP (mm)	4.48 (0.25)	4.43 (0.26)	0.36

*AL* Axial length, *AOD* Angle opening distance, *CT* Corneal thickness, *ELP* Effective lens position, *IOL* Intraocular lens, *LV* Lens vault, *NA* Not available, *post-ACD* Postoperative anterior chamber depth, *pre-ACD* Preoperative anterior chamber depth, *SD* Standard deviation, *SSA* Scleral spur angle, *SSD* Scleral spur distance, *SSW* Scleral spur width, *TISA* Trabecular iris surface area

Significant difference is noted at *P* < 0.05

### Training set and new formula

In training set, preoperative and postoperative SSW showed no significant difference (*P* = 0.994, paired t-test); CT (*P* = 0.004), SSD (*P* = 0.001) and ACD (P < 0.001) showed significant difference between preoperative and postoperative data.

According to the single linear regression analysis in training set, the correlation coefficients with post-ACD were 0.54 for SSD (95% confidence interval [CI], 0.37–0.67; *P* < 0.0001), 0.35 for pre-ACD (95% CI, 0.15–0.52; *P* = 0.0011), 0.34 for AL (95% CI, 0.13–0.51; *P* = 0.0017), 0.19 for SSW (95% CI, − 0.023 – 0.39; *P* = 0.0802), − 0.16 for CT (95%CI, − 0.37 – 0.050; *P* = 0.1318), 0.11 for K (95%CI, − 0.10 – 0.32; *P* = 0.3063), and 0.045 for LV (95%CI, − 0.17 – 0.26; *P* = 0.6802).

Multiple linear regression analysis was used to assess different parameter combinations. Pre-ACD and AL, the combination used by Haigis formula, whose coefficients of determination (R^2^) was 0.19. AL and K used by SRK/T and Holladay 1 formula with an R^2^ of 0.25. The combination of SSD, AL, SSW has the highest R^2^ of 0.49 (Table 2).

**Table 2:** Multiple linear regression analysis of post-ACD in training set (*N* = 85)

	R^2^
Pre-ACD, AL	0.19
AL, K	0.25
SSD	0.29
SSD, AL	0.41
SSD, AL, SSW	0.49
SSD, AL, SSW, Pre-ACD, K	0.49

*AL* Axial length, *K* Corneal power, *post-ACD* Postoperative anterior chamber depth, *pre-ACD* Preoperative anterior chamber depth, *SSD* Scleral spur distance, *SSW* Scleral spur width

Therefore, through multiple linear regression analysis (stepwise method), we develop a new formula to predict post-ACD and to calculate ELP:

post-ACD = 0.192 + 0.867 × SSD + 0.163 × AL – 0.253 × SSW.

ELP predicted by this formula is compared with ELP measured from postoperative AS-OCT image in validation set. To verify our new formula, we also put Holladay 1, SRK/T, Haigis, and a statistical regression formula [16] into comparison.

### Comparison of ELP prediction ability with validation set

**Fig. 3:**
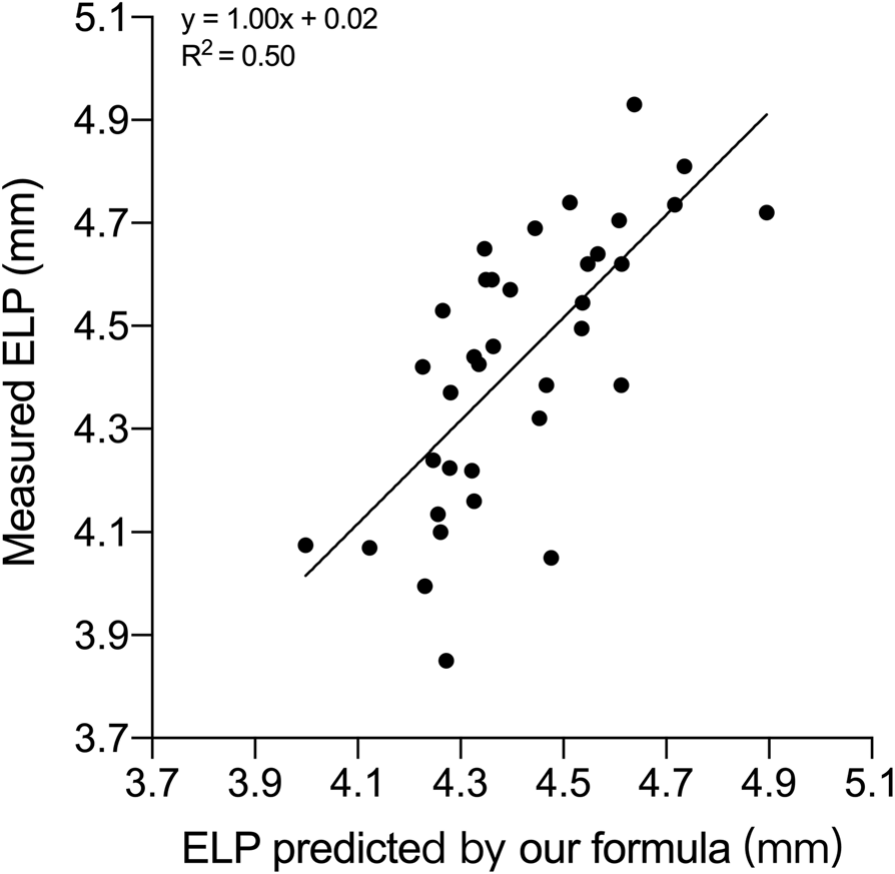
Scatter diagram showing the relativity between measured ELP and ELP predicted by our formula in validation set (*n* = 36)

**Fig. 4:**
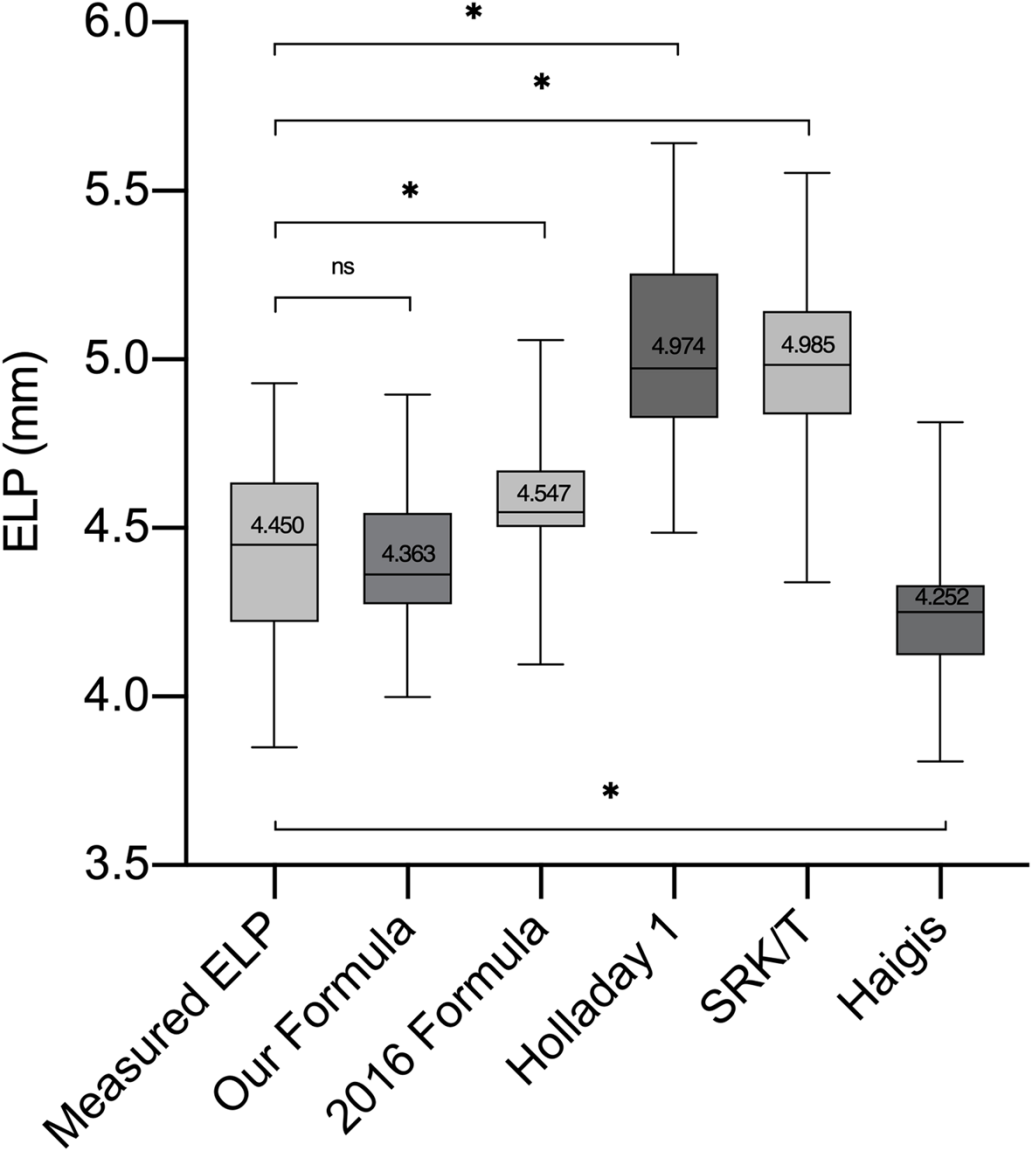
One-way ANOVA analysis in validation set (*N* = 36). Box-and-whisker plot displaying the comparison of Measured Effective Lens Position (ELP) and prediction ELP by our new formula, formula introduced by Goto S et al. in 2016 [16](2016 Formula), Holladay 1, Sanders-Retzlaff-Kraff theoretic (SRK/T) and Haigis formulas. ns = not significant. * = *P* < 0.05

According to one-way ANOVA analysis result (Table 3), our formula showed good compatibility in validation set (Mean absolute error [MAE] = 0.15, R^2^ = 0.50), better than Holladay 1 (MAE = 0.62, R^2^ = 0.12), SRK/T (MAE = 0.58, R^2^ = 0.11), Haigis (MAE = 0.27, R^2^ = 0.06), and the statistic regression formula [16]. (MAE = 0.19, R^2^ = 0.34). The difference between our formula and measured ELP is not significant (*P* = 0.9947, Fig. 3), while other four formulas are significant (Fig. 4).

**Table 3:** One-way analysis of variance and Pearson correlation analysis in validation set (*N* = 36)

	Mean (95% CI)	MAE (SD)	R^2^	*P* value
Measured ELP	4.43 (4.34-4.52)			
New Formula	4.41 (4.35-4.48)	0.15 (0.11)	0.50	0.9947
2016 Formula	4.58 (4.53-4.64)	0.19 (0.18)	0.34	0.0013
Holladay 1	5.04 (4.94-5.14)	0.62 (0.30)	0.12	< 0.0001
SRK/T	5.01 (4.92-5.09)	0.58 (0.30)	0.11	< 0.0001
Haigis	4.24 (4.18-4.30)	0.27 (0.21)	0.06	< 0.0001

*MAE* Mean absolute error, *SD* Standard deviation, *SRK/T* Sanders-Retzlaff-Kraff theoretic, *ELP* Effective lens position, *CI* Confidence interval; 2016 Formula: refers to the formula released in Goto S, et al. [16]

## Discussion

Lens extraction and IOL implantation are widely accepted as the most effective treatment in PACG patients. However, to precisely calculate IOL power in PACG patients is still difficult. Patients who suffer from PACG have characteristic anterior segment such as thick peripheral iris, short AL and ACD [6]. These structural abnormalities make it difficult to predict ELP precisely. Moreover, with the prevalence of MIOL implantation, potential PACG patients could also benefit from accurate prediction to obtain better visual quality and lower risk of angle closure in early stage.

This research focused on PACG patients. The anatomical features of PACG should not be simply regarded as short AL. Previous studies had reported the accuracy of different IOL calculation formulas in cataract patients with different AL [7]. Holladay 1, SRK/T formula performed well in short eye patients. However, our study showed that ELP predicted by those two formulas has significant difference with measured ELP (P < 0.0001) in PACG patients. This result indicated that formulas which only use PCI parameters may not suit PACG patients.

This study aimed to combine AS-OCT parameters with AL to improve the accuracy of IOL power calculation in PACG patients. AS-OCT is a fast, non-invasive and visible examination to analyze anterior chamber [20]. Its quantitative measurement of anterior segment enables more biometric parameters to be available. Compared with pre-ACD which is often affected by ocular diseases and topical drugs, parameters such as SSD and SSW are more stable and valuable in description of ocular anatomic feature. Till now, these parameters could be accuired from AS-OCT only.

Among the 7 parameters we tested (pre-ACD, AL, CT, LV, K, SSD, SSW), multiple linear regression result showed that SSD, AL and SSW play the most important role in the prediction of postoperative ACD. The combination of pre-ACD, AL used in Haigis formula and K, AL used in 3rd generation formula have less correlation coefficient with measured ELP in PACG patients than new parameter combination. This result indicates that the latest generation formula may not be a good choice for PACG patients. First, pre-ACD in PACG patients is misleading to anterior segment analysis. It contributes little to IOL calculation and sometimes even make an error. Second, significant differences have been observed between pre-ACD and post-ACD in PACG patients, which is confirmed in our validation set. This finding indicates that we cannot use pre-ACD to predict post-ACD in PACG patients.

Previous study found that introducing AS-OCT into the prediction of ELP could improve accuracy and decrease postoperative refractory error in cataract patients without PACG [16]. Our study showed that the formula published in 2016 also had better accuracy in PACG patients than Haigis and SRK/T formula. However, PACG has characteristic anterior segment features which demand specially designed formula. This study used SSW rather than pre-ACD to predict ELP and obtain better accuracy than previous studies. Sclera plays a very important role in glaucoma pathophysiology [21]. This research statistically illustrated the relationship between the anatomic structure and clinical practice, indicating that sclera-associated parameters should be introduced into IOL power calculation formula for PACG patients.

There are also some limitations in this study. (1) This study has a small sample size and was conducted at a single-center in China; (2) We didn’t retrieve postoperative refraction error, which is an important postoperative indicator to test whether the prediction is accurate or not [16]; (3) In this research, we focused on ELP based on two considerations. First, postoperative refraction error could be affected by many parameters such as AL and K, even pupil diameter and patient’s condition could slightly distort refractory outcome. Second, postoperative refraction error may change with corneal incision healing. ELP is a crucial parameter directly relate to IOL calculation and more reliable than postoperative refraction error at 1 month after the surgery. The correlation coefficient of our formula is 0.49, which indicated that the mechanism of PACG still remained many questions to be answered. There are also many other factors affect ELP in PACG eyes beyond our knowledge, and more biometric parameters could be measured through AS-OCT [22]; (4) 2016 formula used swept-source OCT and 3-piece IOL, which were different with our study. The variation brought by different OCT device and IOL type should also be considered. Our study used time-domain OCT which is the 1st generation AS-OCT device; nowadays we have more advanced swept-source OCT with higher resolution and acquisition speed to capture biometric image.

In summary, our study showed that using AS-OCT parameters could further improve the accuracy of ELP prediction for PACG patients. Among the 7 parameters we tested, SSD, SSW and AL are most important parameters in ELP prediction. Preoperative ACD makes little contribution to the prediction of postoperative ACD in PACG patients. This research provides a direction for further research in IOL power calculation in PACG patients.

## Data Availability

The datasets used or analyzed during the current study are available from the corresponding author on reasonable request.

## Abbreviations

ANOVA: Analysis of variance
AOD: Angle opening distance
ACD: Anterior chamber depth
AS-OCT: Anterior segment optical coherence tomography
AL: Axial length
CT: Corneal thickness
ELP: Effective lens position
K: Corneal power
IOL: Intraocular lens
LV: Lens vault
MAE: Mean absolute error
MIOL: Multifocal IOL
PAC: Primary angle closure
PACG: Primary angle closure glaucoma
PACS: Primary angle closure suspect
PCI: Partial coherence interferometry
pre-ACD: Preoperative anterior chamber depth
post-ACD: Postoperative anterior chamber depth
SRK/T: Sanders-Retzlaff-Kraff theoretic
SSA: Scleral spur angle
SSD: Scleral spur depth
SSW: Scleral spur width
TISA: Trabecular iris space area

## References

[1] Tham YC, Li X, Wong TY, Quigley HA, Aung T, Cheng CY (2014). Global prevalence of glaucoma and projections of glaucoma burden through 2040: a systematic review and meta-analysis. Ophthalmology.

[2] Quigley HA, Broman AT (2006). The number of people with glaucoma worldwide in 2010 and 2020. Br J Ophthalmol.

[3] Hayashi K, Hayashi H, Nakao F, Hayashi F (2000). Changes in anterior chamber angle width and depth after intraocular lens implantation in eyes with glaucoma. Ophthalmology.

[4] Azuara-Blanco A, Burr J, Ramsay C, Cooper D, Foster PJ, Friedman DS, Scotland G, Javanbakht M, Cochrane C, Norrie J (2016). Effectiveness of early lens extraction for the treatment of primary angle-closure glaucoma (EAGLE): a randomised controlled trial. Lancet (London, England).

[5] Lam DS, Leung DY, Tham CC, Li FC, Kwong YY, Chiu TY, Fan DS (2008). Randomized trial of early phacoemulsification versus peripheral iridotomy to prevent intraocular pressure rise after acute primary angle closure. Ophthalmology.

[6] Sun X, Dai Y, Chen Y, Yu DY, Cringle SJ, Chen J, Kong X, Wang X, Jiang C (2017). Primary angle closure glaucoma: what we know and what we don't know. Prog Retin Eye Res.

[7] Melles RB, Holladay JT, Chang WJ (2018). Accuracy of intraocular Lens calculation formulas. Ophthalmology.

[8] Olsen T (2007). Calculation of intraocular lens power: a review. Acta Ophthalmol Scand.

[9] Campos-Moller X, Ike KAI (2016). Intraocular lens power calculation in primary angle closure. Clin Exp Ophthalmol.

[10] Joo J, Whang WJ, Oh TH, Kang KD, Kim HS, Moon JI (2011). Accuracy of intraocular lens power calculation formulas in primary angle closure glaucoma. Korean J Ophthalmol.

[11] Holladay JT, Prager TC, Chandler TY, Musgrove KH, Lewis JW, Ruiz RS (1988). A three-part system for refining intraocular lens power calculations. J Cataract Refract Surg.

[12] Hoffer KJ (1993). The Hoffer Q formula: a comparison of theoretic and regression formulas. J Cataract Refract Surg.

[13] Retzlaff JA, Sanders DR, Kraff MC (1990). Development of the SRK/T intraocular lens implant power calculation formula. J Cataract Refract Surg.

[14] Haigis W (1993). Occurrence of erroneous anterior chamber depth in the SRK/T formula. J Cataract Refract Surg.

[15] Seo S, Lee CE, Kim YK, Lee SY, Jeoung JW, Park KH (2016). Factors affecting refractive outcome after cataract surgery in primary angle-closure glaucoma. Clin Exp Ophthalmol.

[16] Goto S, Maeda N, Koh S, Ohnuma K, Hayashi K, Iehisa I, Noda T, Nishida K (2016). Prediction of postoperative intraocular Lens position with angle-to-angle depth using anterior segment optical coherence tomography. Ophthalmology.

[17] Nongpiur ME, He M, Amerasinghe N, Friedman DS, Tay WT, Baskaran M, Smith SD, Wong TY, Aung T (2011). Lens vault, thickness, and position in Chinese subjects with angle closure. Ophthalmology.

[18] Goldsmith JA, Li Y, Chalita MR, Westphal V, Patil CA, Rollins AM, Izatt JA, Huang D (2005). Anterior chamber width measurement by high-speed optical coherence tomography. Ophthalmology.

[19] Baikoff G, Jitsuo Jodai H, Bourgeon G (2005). Measurement of the internal diameter and depth of the anterior chamber: IOLMaster versus anterior chamber optical coherence tomographer. J Cataract Refract Surg.

[20] Porporato N, Baskaran M, Aung T (2018). Role of anterior segment optical coherence tomography in angle-closure disease: a review. Clin Exp Ophthalmol.

[21] Pijanka JK, Kimball EC, Pease ME, Abass A, Sorensen T, Nguyen TD, Quigley HA, Boote C (2014). Changes in scleral collagen organization in murine chronic experimental glaucoma. Invest Ophthalmol Vis Sci.

[22] Sng CC, Foo LL, Cheng CY, Allen JC, He M, Krishnaswamy G, Nongpiur ME, Friedman DS, Wong TY, Aung T (2012). Determinants of anterior chamber depth: the Singapore Chinese eye study. Ophthalmology.

